# Low‐dose pembrolizumab in the treatment of advanced non‐small cell lung cancer

**DOI:** 10.1002/ijc.33534

**Published:** 2021-03-06

**Authors:** Jia Li Low, Yiqing Huang, Kenneth Sooi, Yvonne Ang, Zhi Yao Chan, Katie Spencer, Anand Devaprasath Jeyasekharan, Raghav Sundar, Boon Cher Goh, Ross Soo, Wei Peng Yong

**Affiliations:** ^1^ Department of Haematology‐Oncology National University Cancer Institute Singapore (NCIS) Singapore Singapore; ^2^ Department of Pharmacy, National University Hospital National University Health System Singapore Singapore; ^3^ Leeds Institute of Health Sciences, University of Leeds Leeds UK; ^4^ Cancer Science Institute, Singapore (CSI) Singapore Singapore

**Keywords:** Asia, low dose, non‐small cell lung cancer, pembrolizumab

## Abstract

A dose of 200 mg 3‐weekly of pembrolizumab was approved by the Food and Drug Administration (FDA) as treatment for advanced non‐small cell lung cancer (NSCLC) without oncogenic drivers. This is despite evidence showing no difference in efficacy with 2 mg/kg. Our study aimed to assess the efficacy of a lower fixed dose of 100 mg, which is closer to 2 mg/kg weight‐based dose in an average‐sized Asian patient. All patients receiving pembrolizumab for advanced NSCLC from January 2016 to March 2020 in National University Hospital, Singapore, were included in this retrospective observational study. The effect of pembrolizumab 100 mg (Pem100) vs 200 mg (Pem200) upon survival outcomes, toxicity and cost were examined. One hundred fourteen patients received pembrolizumab. Sixty‐five (57%) and 49 (43%) received Pem100 and Pem200, respectively. There was no difference in progression‐free survival (PFS) and overall survival (OS) between Pem100 vs Pem200 as a single agent (PFS: 6.8 vs 4.2 months, hazard ratio [HR] 0.72, 95% confidence interval [CI] 0.36‐1.46, *P* = .36; 9 month OS: 58% vs 63%, HR 1.08, 95% CI 0.48‐2.41, *P* = .86) and when combined with chemotherapy (9‐month PFS: 60% vs 50%, HR0.84, 95% CI 0.34‐2.08, *P* = .71; 9‐month OS: 85% vs 58%, HR 0.27, 95% CI 0.062‐1.20, *P* = .09). No significant difference in response rate or ≥G3 immune‐related toxicities between Pem100 and Pem200 was observed. A cost minimisation analysis evaluating the degree of cost savings related to drug costs estimated a within study cost saving of SGD4,290,912 and cost saving per patient of SGD39,942 in the Pem100 group. A 100 mg of pembrolizumab appears to be effective with reduction in cost. A randomised trial should be done to investigate a lower dose of pembrolizumab.

## Abstract 2

What's new?

Pembrolizumab, a monoclonal antibody directed against the PD‐1 receptor, has received FDA approval for the treatment of lung cancer at a fixed dose of 200 mg every 3 weeks. However, doses above 2 mg/kg show a lack of benefit, calling for further evaluation in Asian populations. This retrospective observational study demonstrates the efficacy of a lower fixed dose of pembrolizumab (100 mg every 3 weeks) compared with standard‐dose pembrolizumab. The results also confirm the clinical activity of pembrolizumab at a lower dose than 2 mg/kg every 3 weeks, which could provide considerable cost savings to patients and the health system.

**Abbreviations**
AE: adverse event
DCR: disease control rate
FDA: Food and Drug Administration
HR: hazard ratio
irAEs: immune‐related adverse events
NSCLC: non‐small cell lung cancer
OS: overall survival
PD: progressive disease
PD‐1: programmed cell death protein 1
Pem100: pembrolizumab 100 mg 3‐weekly
Pem200: pembrolizumab 200 mg 3‐weekly
PFS: progression‐free survival
PR: partial response
RR: response rate
SD: stable disease
TPS: tumour proportion score

## 1 INTRODUCTION

Worldwide, lung cancer is the most common cancer and leading cause of cancer death.^1^ Cancer immunology has enabled the development of immune modulators that have markedly altered the treatment landscape for patients with advanced non‐small cell lung cancers (NSCLC) that do not harbour oncogenic drivers.^2^

Pembrolizumab is a fully humanised immunoglobulin G4 monoclonal antibody directed against the programmed cell death protein 1 (PD‐1) receptor, antagonising the interaction between itself and its ligand, resulting in anti‐tumour immune response.^3^, ^4^, ^5^ Since the Food and Drug Administration (FDA) approval of pembrolizumab in 2015^6^ in pre‐treated NSCLC,^7^, ^8^, ^9^, ^10^ pembrolizumab has rapidly transited to standard of care first line treatment as a monotherapy^11^, ^12^, ^13^ or with chemotherapy^14^, ^15^, ^16^, ^17^ for NSCLC with no oncogenic drivers.

In the landmark KEYNOTE‐001, an ex vivo pharmacokinetics study of PD‐1 receptor saturation found complete peripheral target engagement at 1 mg/kg. In the initial dose escalation cohort, durable anti‐tumour activity across all patient cohorts from 1‐10 mg/kg once every 3 weeks was observed.^18^ The subsequent expansion cohorts demonstrated that the efficacy of 2 mg/kg 3‐weekly was similar to higher dose 10 mg/kg 2‐weekly regimen but a dose of <2 mg/kg was not examined.^7^, ^9^, ^12^, ^19^

Despite the pharmacokinetics profile showing PD‐1 receptor saturation at 1 mg/kg and clinical efficacy at 2 mg/kg, a flat dose of 200 mg 3‐weekly was used in Phase III trials leading to the FDA approval of 200 mg every 3 weeks.^14^, ^15^, ^16^, ^17^ More recently, the FDA granted accelerated approval of a new dosing regimen of 400 mg every 6 weeks.^6^

Goldstein et al performed an economic analysis comparing the FDA approved fixed dosing and personalised dosing at 2 mg/kg and estimated a cost saving of USD$0.8 billion annually to the United States healthcare system based on an average weight of 75 kg in an American adult patient.^20^, ^21^, ^22^, ^23^ The average weight of an Asian patient is 60 kg giving an average personalised dose of 120 mg in this population.^24^

Given the lack of benefit demonstrated by pembrolizumab at doses above 2 mg/kg, the lower weight of Asian patients, economic benefits of a lower dose and packaging of pembrolizumab in 100 mg vials, a fixed dose of 100 mg pembrolizumab required evaluation in an Asian population.

Using a retrospective observational design, our study aimed to evaluate the efficacy of low‐dose pembrolizumab (Pem100) compared with standard‐dose pembrolizumab (Pem200) in the treatment of NSCLC.

## 2 METHODS

### 2.1 Patients and treatment

All patients receiving palliative intent pembrolizumab for advanced NSCLC with or without chemotherapy between January 2016 and March 2020 in an academic tertiary medical centre (National University Hospital, Singapore) were identified retrospectively from the pharmaceutical database. Baseline patient demographics, tumour and treatment characteristics were extracted from the electronic medical records.

The dose of 100 mg was routinely delivered based on an approximate 2 mg/kg weight‐based dose, for patients who did not have an adequate financial reimbursement plan or based on physician's preference. Local protocols continue treatment until disease progression, unacceptable toxicities, death, patient's decision to stop treatment or after a total of 35 cycles of pembrolizumab although some patients who remained progression free after 35 cycles continued treatment.

### 2.2 Response evaluation

Chest and/or abdominal CT scans were performed by clinicians every 8‐12 weeks as part of routine clinical care, to evaluate patient's response and assess for disease progression. The scans were evaluated by investigators retrospectively. In line with the KEYNOTE studies efficacy analysis was examined only in patients without an oncogenic driver mutation. A systemic response to pembrolizumab was measured by standard Response Evaluation Criteria in Solid Tumours (V.1.1).^25^ The best response was classified as progressive disease (PD), stable disease (SD), partial response (PR) and complete response. Progression‐free survival (PFS) was measured from time of initiation of drug to disease progression by RECIST or death due to any cause. Overall survival (OS) was measured from time of initiation of drug to death. Safety analysis examined the incidence of ≥ Grade 3 immune‐related adverse events (irAEs) and adverse events (AEs) as recorded by clinicians.

### 2.3 IHC of PDL1

The PD‐L1 IHC 22C3 pharmDx assay^26^, ^27^ was used to assess PD‐L1 expression in formalin‐fixed tumour samples obtained at the time of diagnosis of metastatic disease. PD‐L1 clone 22C3 is from Dako and stained on Roche Ventana Benchmark Ultra ISH/IHC autostainer with Optiview Polymer Detection Kit. Interpretation of PD‐L1 expression is based on the interpretation guide provided by Dako and was characterised according to tumour proportion score (TPS).^28^

### 2.4 Statistical and economic analysis

Differences in the baseline characteristics of patients receiving Pem100 and Pem200 were evaluated using the Fisher's exact test. Survival analyses were performed using the Kaplan‐Meier method and were compared using a log‐rank test. Multi‐variable Cox proportional hazard regression models were used to assess the relationship between baseline factors (including treatment) and survival. A *P*‐value of <.05 was considered statistically significant. All statistical tests were two‐sided and were performed using IBM SPSS Statistics Version 22.

Based on an acceptance of non‐inferior survival and toxicity outcomes, a limited economic evaluation was carried out using a cost‐minimisation approach.^29^ This assessed the monetary savings available from the use of Pem100 instead of Pem200 based on the total and median cycles of pembrolizumab received by the study population and the price of a 100 mg vial of pembrolizumab. Sensitivity analysis considered the potential savings within the study population if all patients and if patients weighing ≤100 kg (translating to a dose of at least 1 mg/kg) were to receive Pem100. Given the identical regimens and observed clinical outcomes, all other costs were assumed to remain constant.

## 3 RESULTS

### 3.1 Patient characteristics

One hundred fourteen patients received pembrolizumab for advanced NSCLC from January 2016 to March 2020 in National University Hospital (Singapore). Baseline demographics are shown in Table 1. Median age was 67.4 years (range, 28.4‐92.2). A majority of patients were male (86, 75%), Chinese (83, 73%), former/current smokers (81, 71%) and had an ECOG status of 0/1 (88, 77%). The average weight was 59 kg (range, 31‐103).

**TABLE 1:** Baseline demographics

		Total (n = 114)	Pem 200 (n = 49)	Pem 100 (n = 65)	*P* values
Age at diagnosis (median, range)		67.4 (28.4‐92.2)	60.5 (28.4‐80.0)	69.9 (42.8‐92.2)	<.001
Weight (median, range)		59 (31‐103)	59 (37‐103)	59 (31‐101)	.245
Sex	Male	86 (75%)	41 (84%)	45 (69%)	.084
	Female	28 (25%)	8 (16%)	20 (31%)	
Ethnicity	Chinese	83 (73%)	32 (65%)	51 (78%)	.006
	Malay	17 (15%)	6 (12%)	11 (17%)	
	Indian	1 (1%)	0 (0%)	1 (2%)	
	Others	13 (11%)	11 (23%)	2 (3%)	
Smoking history	Current/ex‐smoker	81 (71%)	32 (65%)	49 (75%)	.298
	Never smoker	33 (29%)	17 (35%)	16 (25%)	
Performance status	0‐1	88 (77%)	42 (86%)	46 (71%)	.053
	2	11 (10%)	1 (2%)	10 (15%)	
	≥3	14 (12%)	6 (12%)	8 (12%)	
	Unknown	1 (1%)	0 (0%)	1 (2%)	
Renal function	CrCl <30	2 (2%)	1 (2%)	1 (2%)	<.001
	CrCl 30 to <40	6 (5%)	0 (0%)	6 (9%)	
	CrCl 40 to <50	7 (6%)	2 (4%)	5 (8%)	
	CrCl 50 to <60	23 (20%)	3 (6%)	20 (31%)	
	CrCl >60	76 (67%)	43 (88%)	33 (51%)	
Hepatic function	Liver dysfunction	6 (5%)	3 (6%)	3 (5%)	.519

Tumour characteristics are summarised in Table 2. Eighty‐one (71%), 13 (11%) and 16 (14%) of patients had adenocarcinoma, squamous cell carcinoma and poorly differentiated carcinoma, respectively. Sixty‐three (55%), 31 (27%) and 16 (14%) of patients had a PDL1‐TPS score of ≥50%, 1%‐49% and 0%, respectively. Pembrolizumab was prescribed as first‐line therapy in 91 (80%), second‐line therapy in 15 (13%), third line and beyond in 8 (7%) patients. Sixty‐five (57%) and 49 (43%) received pembrolizumab as monotherapy and combined with chemotherapy, respectively.

**TABLE 2:** Tumour and treatment characteristics

		Total (n = 114)	Pem200 (n = 49)	Pem100 (n = 65)	*P* values
Histological subtype	Adenocarcinoma	81 (71%)	37 (76%)	44 (68%)	.553
	Squamous cell carcinoma	13 (11%)	7 (14%)	6 (9%)	
	Poorly differentiated	16 (14%)	5 (10%)	11 (17%)	
	Others	4^a^ (4%)	0 (0%)	4 (6%)	
PD‐L1 TPS score	0%	16 (14%)	11 (22%)	5 (8%)	.005
	1%‐49%	31 (27%)	18 (37%)	13 (20%)	
	≥50%	63 (55%)	19 (39%)	44 (68%)	
	Unknown	4 (4%)	1 (2%)	3 (4%)	
Driver mutation status	*EGFR* positive	7 (6%)	2 (4%)	5 (8%)	1.00
	*ALK* positive	1 (1%)	0 (0%)	1 (2%)	
	*ROS* positive	2 (2%)	2 (4%)	0 (0%)	
Line of treatment in palliative setting	First line	91 (80%)	43 (88%)	48 (74%)	.223
	Second line	15 (13%)	4 (8%)	11 (17%)	
	Third line and beyond	8 (7%)	2 (4%)	6 (9%)	
Partner drug, n (%)	Monotherapy	65 (57%)	17 (35%)	48 (74%)	<.001
	Combined with chemotherapy	49^b^ (43%)	32 (65%)	17 (26%)	
Dose/kg of pembrolizumab	Median dose received (range)	2.27 (1.24‐4.98)	2.87 (1.94‐4.98)	1.85 (1.24‐3.2)	<.001
	Number of patients who received <2 mg/kg	42	2 (4%)	40 (62%)	<.001

^a^ Adenosquamous (n = 1), epithelioma‐like (1), pleomorphic (1), unknown (1).

^b^ Chemotherapy combination—carboplatin/pemetrexed (n = 44), carboplatin/paclitaxel (n = 3), carboplatin/abraxane (n = 2).

### 3.2 Pembrolizumab dosing

A majority of patients (65/114, 57%) received pembrolizumab at a starting dose of 100 mg 3‐weekly (Pem100). The remaining 43% received pembrolizumab at a starting dose of 200 mg 3‐weekly (Pem200). The two cohorts were matched in terms of gender, smoking history, ECOG status and histological subtype (*P* > .05). Patients in the Pem200 group were younger (median age 60.5 vs 69.9, *P* < .001), with worse renal function (*P* < .001) compared to those in the Pem100 group (Table 1).

Significantly more patients in Pem100 had a PD‐L1 (TPS) score of ≥50% (68% vs 39%, *P* = .005) and received pembrolizumab as a monotherapy (74% vs 35%, *P* < .001). Median dose received was 2.87 mg/kg vs 1.85 mg/kg (*P* < .001) and a <2 mg/kg dose was received by 4% vs 62% (*P* < .001) in the Pem200 and Pem100, respectively.

### 3.3 Outcomes

#### 3.3.1 Survival

Ten patients with oncogenic driven NSCLC who received pembrolizumab were excluded from the analysis. Median duration of follow‐up was 14.8 months.

**FIGURE 1:**
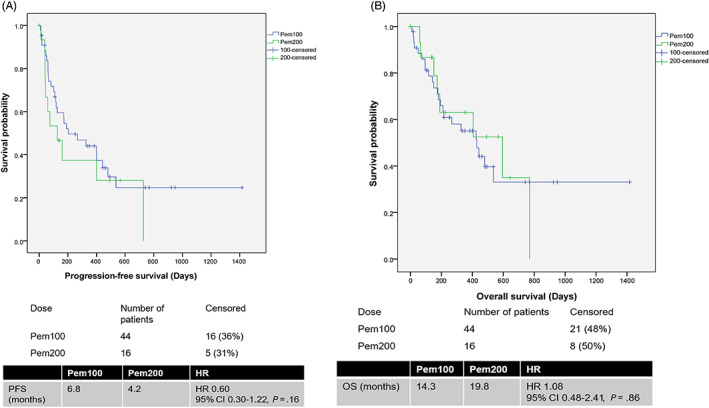
A, Progression free survival of single agent Pem100 and Pem200. B, Overall survival of single agent Pem100 and Pem200 [Color figure can be viewed at wileyonlinelibrary.com]

**FIGURE 2:**
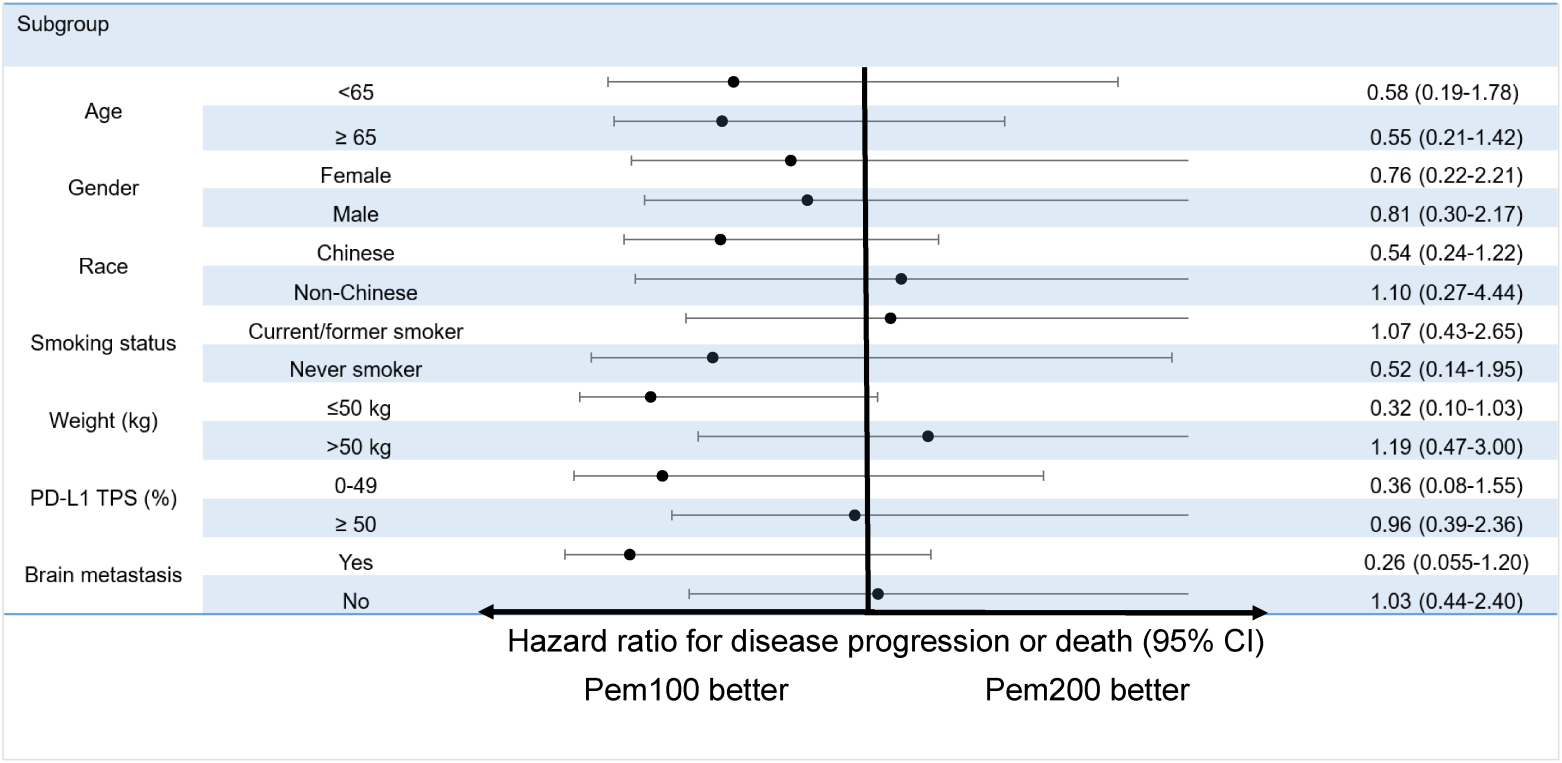
Multivariable cox‐proportional hazards model for progression free survival of patients receiving pembrolizumab monotherapy [Color figure can be viewed at wileyonlinelibrary.com]

**FIGURE 3:**
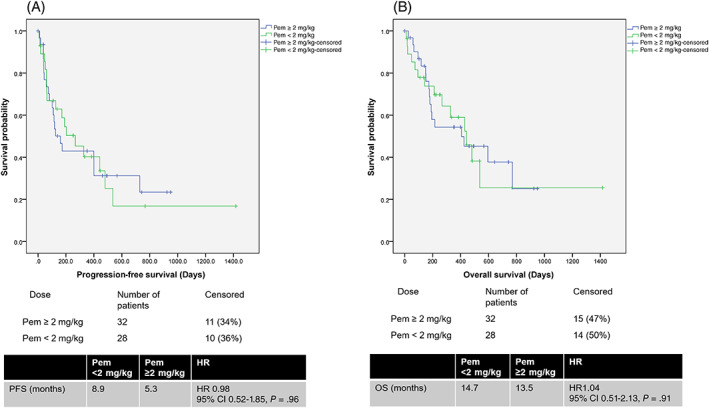
A, Progression free survival of single agent pembrolizumab ≥2mg/kg and pembrolizumab <2mg/kg. B, Overall survival of single agent pembrolizumab ≥2mg/kg and pembrolizumab <2mg/kg [Color figure can be viewed at wileyonlinelibrary.com]

The median PFS of Pem100 vs Pem200 as a single agent was not statistically significant at 6.8 vs 4.2 months (HR 0.60, 95% CI 0.30‐1.22, *P* = .16) (Figure 1A ). PFS for Pem100 and Pem200 did not differ in all subgroups examined (Figure 2). Median OS was 14.3 vs 19.8 months (HR 1.08, 95% CI 0.48‐2.41, *P* = .86) for Pem100 vs Pem200, respectively (Figure 1B). Median PFS and OS of pembrolizumab <2 vs ≥2 mg/kg as a single agent did not differ (PFS: 8.9 vs 5.3 months HR 0.98, 95% CI 0.52‐1.85, *P* = .96; OS 14.7 vs 13.5 months, HR 1.04, 95% CI 0.51‐2.13, *P* = .91) (Figure 3A,B).

Where Pembrolizumab was delivered with chemotherapy, the median PFS was not reached in the Pem100 cohort vs 11.9 months (HR 0.84, 95% CI 0.34‐2.08, *P* = .71) for the Pem200 cohort. Median OS was not reached in either group. Nine‐month OS with chemotherapy was 85% vs 58% (HR 0.27, 95% CI 0.062‐1.20, *P* = .09) for Pem100 and Pem200, respectively.

### 3.4 Response rates

We analysed the response rates (RRs) of 88 patients who received pembrolizumab in the first line setting. Forty‐six received pembrolizumab as a single agent and 42 received pembrolizumab combined with chemotherapy. RR and disease control rate (DCR) were numerically higher in patients who received single agent Pem100 compared to Pem200 (RR: 45.5% vs 23.1%, *P* = .20, CBR: 72.2% vs 53.8%, *P* = .30). This was however not statistically significant. Similarly, when pembrolizumab was combined with chemotherapy, there was also no difference in the RR and DCR (RR 46.1% vs 48.3%, *P* = 1.00, CBR 92% vs 86, *P* = 1.00 for Pem100 and Pem200, respectively) (Table 3).

**TABLE 3:** Response outcomes of Pem100 vs Pem200 in first line setting

	Single agent (n = 46)			Combined with chemotherapy (n = 42)		
	Pem 100 (n = 33)	Pem 200 (n = 13)	*P* value	Pem 100 (n = 13)	Pem 200 (n = 29)	*P* value
Progressive disease	8 (24%)	4 (31%)		1 (7%)	2 (7%)	
Stable disease	9 (27%)	4 (31%)		6 (46%)	11 (38%)	
Partial response	14 (42%)	3 (23%)		5 (38%)	14 (48%)	
Complete response	1 (3%)	0 (0%)		1 (8%)	0 (0%)	
Not evaluable	1 (3%)	2 (15%)		0 (0%)	2 (7%)	
Response rate, n (%)	15 (45.5%)	3 (23.1%)	.20	6 (46.1%)	14 (48.3%)	1.00
Disease control rate^a^, n (%)	24 (72.7%)	7 (53.8%)	.30	12 (92%)	25 (86%)	1.00

^a^ Disease control rate = stable disease + partial response + complete response.

### 3.5 Toxicities

Eighteen patients discontinued treatment due to toxicities. There was no dose relationship between pembrolizumab and serious irAEs. The rates of G3 or more irAEs between Pem100 and Pem200 were observed to be 17% vs 22%, *P* = .5.

### 3.6 Cost analysis

A 100 mg vial of pembrolizumab costs SGD5706 (1 SGD ≈ 0.72 USD) in Singapore. The total number of cycles received by all the patients in our study is 1243, with 752 vs 491 cycles delivered in the Pem100 and Pem200 group, respectively. The median number of cycles was 7 (range, 1‐70 cycles).

We estimated a total cost saving in the study population of SGD 4,290 912 based on the total number of cycles of pembrolizumab received by the Pem100 group. Assuming Pem100 was used instead of Pem200 across the entire study population, the cost minimisation analysis demonstrates a cost saving of SGD 7, 092, 558 and SGD 39942 per patient based on the median number of cycles received in our study population. A further sensitivity analysis of Pem100 in patients weighing ≤100 kg demonstrates a total cost savings of SGD 70065706.

## 4 DISCUSSION

To our knowledge, our study represents the largest cohort to date in which the efficacy of a lower fixed dose of 100 mg pembrolizumab given 3‐weekly is demonstrated.^30^ We also confirm the clinical activity of pembrolizumab at a dose lower than 2 mg/kg 3‐weekly.

Many studies have demonstrated that dose selection of immune checkpoint inhibitors can be challenging with non‐linear relationships between dose and clinical outcomes. A pharmacokinetic analysis of doses of 200 mg and 2 mg/kg showed similar exposure distributions with no advantage to either dosing approach.^31^ Similarly, modelling of data from KEYNOTE‐001 demonstrated that pembrolizumab kinetics are linear above 0.3 mg/kg and there is 95% trough target engagement with dosing at 0.8 mg/kg 3‐weekly with saturation of PD‐L1 receptor at a dose ≥1 mg/kg.^32^, ^33^, ^34^ Indeed, the expansion cohort of KEYNOTE‐001 demonstrated clinical efficacy at its lowest evaluated dose of 2 mg/kg.^19^ Other dosing strategies at <2 mg/kg, dose banding and increasing the interval of dosing have also demonstrated efficacy.^35^, ^36^ The Canadian Agency of Drug and Technologies used a pharmacokinetic model demonstrating adequate trough PD‐1 target engagement of 96% for those weighing 150 kg receiving the 400 mg 6‐weekly.^37^, ^38^, ^39^

In our study, the median patient weight was 59 kg. More than half of our patients received a fixed dose of 100 mg, with a median dose of 1.85 mg/kg (range, 1.24‐3.2 mg/kg 3‐weekly) in the Pem100 group. This was close to a dose of 2 mg/kg and could explain why we did not see a difference in the efficacy in Pem100 vs Pem200. No efficacy difference between <2 and ≥ 2 mg/kg 3‐weekly. In fact, survival results numerically favoured <2 mg/kg.

A fixed 100 mg dose of pembrolizumab appears to be cost efficient and logistically feasible, requiring a complete vial per patient. Goldstein et al demonstrated huge cost savings to the US healthcare system by using a personalised dosing of 2 mg/kg.^20^ In fact, the economic impact may be underestimated given the rising price of pembrolizumab.^40^ Despite this, weight‐based dosing is not widely adopted. With pembrolizumab packaged and sold as 100 mg vials by pharmaceutical companies in many countries, weight‐based dosing is logistically challenging. Furthermore, vial sharing is not widely adopted as vial misuse, including unsafe handling, has led to vial contamination and risks of bloodborne illness transmission between patients.^21^ The results of our study provide a practical solution and eliminate the need for vial sharing.

Our study has its limitations. Despite the attractiveness of dosing pembrolizumab at a 100 mg fixed dose 3‐weekly or 200 mg 6‐weekly, fixed dosing must be interpreted with caution in a heavier patient. None of the patients in our study received a dose a <1 mg/kg, with a dose range of 1.24 to 1.99 mg/kg for patients who received <2 mg/kg pembrolizumab. Cross‐trial comparisons of our study against the other KEYNOTE studies are not valid given the different nature of the study design and heterogenicity of our study population. Moreover, the retrospective nature of the study, differing baseline characteristics and limited sample size does not allow for valid efficacy comparison among different dosing strategies. Also, there are some imbalances in our study groups with more patients in the Pem100 group having a PDL1 TPS score ≥50%. In a non‐randomised setting, this may result in selection bias, potentially accounting for the favourable outcome observed here for Pem100. However, most of these patients also received pembrolizumab as monotherapy and the subgroup analysis of PFS based on PDL1 expression in patients receiving pembrolizumab monotherapy did not differ. Thirdly, the relatively limited sample size limits the power of our study to demonstrate a statistically significant difference. Finally, given no difference was identified in the clinical outcomes of the two regimens a cost minimisation analysis was used to examine the cost saving provided by Pem100. This was not planned a priori and simply provides an indication of possible savings. The costs assessed are only those of the drug and do not include regimen related costs such as drug administration, premedication, clinic visits, subsequent therapy and AE management. Based on the study outcomes these costs are not anticipated to vary; however, further formal assessment of the cost‐utility of Pem100 should be considered alongside any future randomised study.

Despite these limitations, our study is the first to suggest clinical efficacy of pembrolizumab at a fixed dose of 100 mg 3‐weekly. This lower dose could be efficacious and provide considerable cost savings to both patients and the health system more widely. Such savings could be redistributed to other health needs.^41^, ^42^, ^43^

## 5 CONCLUSION

In our study, pembrolizumab had efficacy at a dose of 100 mg 3‐weekly. With the expanding role of immune checkpoint inhibitors in many tumour types, the principles and solutions discussed here will be highly relevant to oncologists, policymakers and patients alike. A randomised prospective trial is now required to further investigate the role and cost‐effectiveness of lower‐fixed dosing of pembrolizumab at 100 mg 3‐weekly or 200 mg 6‐weekly.

## CONFLICT OF INTEREST

A. Jeyasekharan has received consultancy fees from Turbine Ltd, Astra Zeneca, Janssen and MSD along with travel funding from Perkin Elmer and research funding from Janssen. R. Sundar is advisory board for Bristol Myers Squibb, Merck, Eisai, Bayer, Taiho, Novartis and MSD, and has received Honararia for talks from MSD, Eli Lily, BMS, Roche, Taiho and Astra Zeneca, travel funding from Roche, Astra Zeneca, Taiho and Eisai and research funding from Paxman Coolers, MSD. B. C. Goh has received research funding from Merck and Co, Bayer Healthcare, Taiho Pharmaceuticals, Squibb and Adagene, consultancy role in Adagene, Bayer Healthcare, Merck and co and Astra Zeneca, and has stock ownership in Gilead sciences, Moderna Inc. R. Soo has received honorarium from Astra Zeneca, Amgen, Bayer, BMS, Boehringer Ingelheim, Lilly, Merck, Novartis, Pfizer, Roche, Taiho, Takeda and Yuhan. W. P. Yong has received honorarium advisory board and speaker fee from Taihio, Ipsen, Novartis, Eisai, BMS, SD, Sanofi‐Aventis, Bayer, Astra Zeneca and royalty from miRXES.

## ETHICS STATEMENT

The study was approved by the National Health Group Domain Specific Review Board (NHG DSRB) (Reference number: 2017/012654) and was conducted in accordance to the Declaration of Helsinki provision. Informed consent was waived because of the retrospective design of the study.

## Data Availability

The data that support the findings of this study are available on request from the corresponding author.

## References

[1] Bray F , Ferlay J , Soerjomataram I , Siegel RL , Torre LA , Jemal A . Global cancer statistics 2018: GLOBOCAN estimates of incidence and mortality worldwide for 36 cancers in 185 countries. CA Cancer J Clin. 2018;68:394‐424. 30207593 10.3322/caac.21492

[2] Low JL , Walsh RJ , Ang Y , Chan G , Soo RA . The evolving immuno‐oncology landscape in advanced lung cancer: first‐line treatment of non‐small cell lung cancer. Ther Adv Med Oncol. 2019;11:175883591987036. 10.1177/1758835919870360 PMC6716180 31497071

[3] Sharpe AH , Freeman GJ . The B7‐CD28 superfamily. Nat Rev Immunol. 2002;2:116‐126. 11910893 10.1038/nri727

[4] Iwai Y , Ishida M , Tanaka Y , Okazaki T , Honjo T , Minato N . Involvement of PD‐L1 on tumor cells in the escape from host immune system and tumor immunotherapy by PD‐L1 blockade. Proc Natl Acad Sci USA. 2002;99:12293‐12297. 12218188 10.1073/pnas.192461099 PMC129438

[5] Keir ME , Butte MJ , Freeman GJ , Sharpe AH . PD‐1 and its ligands in tolerance and immunity. Annu Rev Immunol. 2008;26:677‐704. 18173375 10.1146/annurev.immunol.26.021607.090331 PMC10637733

[6] Pai‐Scherf L , Blumenthal GM , Li H , et al. FDA approval summary: pembrolizumab for treatment of metastatic non‐small cell lung cancer: first‐line therapy and beyond. Oncologist. 2017;22:1392‐1399. 28835513 10.1634/theoncologist.2017-0078 PMC5679831

[7] Garon EB , Rizvi NA , Hui R , et al. Pembrolizumab for the treatment of non‐small‐cell lung cancer. N Engl J Med. 2015;372:2018‐2028. 25891174 10.1056/NEJMoa1501824

[8] Herbst RS , Baas P , Kim DW , et al. Pembrolizumab versus docetaxel for previously treated, PD‐L1‐positive, advanced non‐small‐cell lung cancer (KEYNOTE‐010): a randomised controlled trial. Lancet. 2016;387:1540‐1550. 26712084 10.1016/S0140-6736(15)01281-7

[9] Kang SP , Gergich K , Lubiniecki GM , et al. Pembrolizumab KEYNOTE‐001: an adaptive study leading to accelerated approval for two indications and a companion diagnostic. Ann Oncol. 2017;28:1388‐1398. 30052728 10.1093/annonc/mdx076 PMC5452070

[10] Garon EB , Hellmann MD , Rizvi NA , et al. Five‐year overall survival for patients with advanced non‐small‐cell lung cancer treated with pembrolizumab: results from the phase I KEYNOTE‐001 study. J Clin Oncol. 2019;37:2518‐2527. 31154919 10.1200/JCO.19.00934 PMC6768611

[11] Reck M , Rodríguez‐Abreu D , Robinson AG , et al. keynote 024. N Engl J Med. 2016;375:1823‐1833. 10.1056/NEJMoa1606774 27718847

[12] Reck M , Rodríguez‐Abreu D , Robinson AG , et al. Updated analysis of KEYNOTE‐024: Pembrolizumab versus platinum‐based chemotherapy for advanced non–small‐cell lung cancer with PD‐L1 tumor proportion score of 50% or greater. J Clin Oncol. 2019;37:537‐546. 30620668 10.1200/JCO.18.00149

[13] Mok TSK , Wu YL , Kudaba I , et al. Pembrolizumab versus chemotherapy for previously untreated, PD‐L1‐expressing, locally advanced or metastatic non‐small‐cell lung cancer (KEYNOTE‐042): a randomised, open‐label, controlled, phase 3 trial. Lancet. 2019;393:1819‐1830. 30955977 10.1016/S0140-6736(18)32409-7

[14] Langer CJ , Gadgeel SM , Borghaei H , et al. Carboplatin and pemetrexed with or without pembrolizumab for advanced, non‐squamous non‐small‐cell lung cancer: a randomised, phase 2 cohort of the open‐label KEYNOTE‐021 study. Lancet Oncol. 2016;17:1497‐1508. 27745820 10.1016/S1470-2045(16)30498-3 PMC6886237

[15] Gandhi L , Rodríguez‐Abreu D , Gadgeel S , et al. Pembrolizumab plus chemotherapy in metastatic non‐small‐cell lung cancer. N Engl J Med. 2018;378:2078‐2092. 29658856 10.1056/NEJMoa1801005

[16] Gadgeel S , Rodríguez‐Abreu D , Speranza G , et al. Updated analysis from KEYNOTE‐189: pembrolizumab or placebo plus pemetrexed and platinum for previously untreated metastatic nonsquamous non‐small‐cell lung cancer. J Clin Oncol. 2020;38:1505‐1517. 32150489 10.1200/JCO.19.03136

[17] Paz‐Ares L , Luft A , Vicente D , et al. Pembrolizumab plus chemotherapy for squamous non‐small‐cell lung cancer. N Engl J Med. 2018;379:2040‐2051. 30280635 10.1056/NEJMoa1810865

[18] Chatterjee M , Elassaiss‐Schaap J , Lindauer A , et al. Population pharmacokinetic/pharmacodynamic modeling of tumor size dynamics in pembrolizumab‐treated advanced melanoma. CPT Pharmacometrics Syst Pharmacol. 2017;6:29‐39. 27896938 10.1002/psp4.12140 PMC5270297

[19] Hui R , Garon EB , Goldman JW , et al. Pembrolizumab as first‐line therapy for patients with PD‐L1‐positive advanced non‐small cell lung cancer: a phase 1 trial. Ann Oncol. 2017;28:874‐881. 28168303 10.1093/annonc/mdx008 PMC6354672

[20] Goldstein DA , Gordon N , Davidescu M , et al. A phamacoeconomic analysis of personalized dosing vs fixed dosing of pembrolizumab in firstline PD‐L1‐positive non‐small cell lung cancer. J Natl Cancer Inst. 2017;109(11). 10.1093/jnci/djx063 29059432

[21] Perz JF , Thompson ND , Schaefer MK , Patel PR . US outbreak investigations highlight the need for safe injection practices and basic infection control. Clin Liver Dis. 2010;14:137‐151. 20123446 10.1016/j.cld.2009.11.004

[22] Abeysinghe T , Lim J . Singapore's healthcare financing: some challenges. Singapore's Health Care Finance; 2010.

[23] Bai Y , Shi C , Li X , Liu F . Healthcare System in Singapore. Colombo University; 2012.

[24] Walpole SC , Prieto‐Merino D , Edwards P , Cleland J , Stevens G , Roberts I . The weight of nations: an estimation of adult human biomass. BMC Public Health. 2012;12:439. 22709383 10.1186/1471-2458-12-439 PMC3408371

[25] Schwartz LH , Litière S , De Vries E , et al. RECIST 1.1 ‐ update and clarification: from the RECIST committee. Eur J Cancer. 2016;62:132‐137. 27189322 10.1016/j.ejca.2016.03.081 PMC5737828

[26] Naiyer A , Edward B , Natasha L , et al. Optimizing PDL1 as a biomarker of response with pembrolizumab (pembro; MK‐3475) as first‐line therapy for PDL1‐positive metastatic non‐small cell lung cancer (NSCLC): updated data from KEYNOTE‐001. J Clin Oncol. 2015;33, no. 15_suppl: 8026‐8026.

[27] Ilie M , Khambata‐Ford S , Copie‐Bergman C , et al. Use of the 22C3 anti–PD‐L1 antibody to determine PD‐L1 expression in multiple automated immunohistochemistry platforms. PLoS One. 2017;12(8):e0183023. 10.1371/journal.pone.0183023 PMC5552229 28797130

[28] Roach C , Zhang N , Corigliano E , et al. Development of a companion diagnostic PD‐L1 immunohistochemistry assay for pembrolizumab therapy in non‐small‐cell lung cancer. Appl Immunohistochem Mol Morphol. 2016;24:392‐397. 27333219 10.1097/PAI.0000000000000408 PMC4957959

[29] Wailoo A , Dixon S . The use of cost minimisation analysis for the appraisal of health technologies. NICE Decision Support Unit; May 2019. http://nicedsu.org.uk/wp-content/uploads/2020/11/Cost-minimisation-final-DSU-report-10-5-19_final.pdf

[30] Yoo SH , Keam B , Kim M , et al. Low‐dose nivolumab can be effective in non‐small cell lung cancer: alternative option for financial toxicity. ESMO Open. 2018;3:e000332. 30094065 10.1136/esmoopen-2018-000332 PMC6069908

[31] Freshwater T , Kondic A , Ahamadi M , et al. Evaluation of dosing strategy for pembrolizumab for oncology indications. J Immunother Cancer. 2017;5:43. 28515943 10.1186/s40425-017-0242-5 PMC5433037

[32] Elassaiss‐Schaap J , Rossenu S , Lindauer A , et al. Using model‐based “learn and confirm” to reveal the pharmacokinetics‐pharmacodynamics relationship of Pembrolizumab in the KEYNOTE‐001 trial. CPT Pharmacometrics Syst Pharmacol. 2017;6(1):21‐28. 10.1002/psp4.12132 PMC5270295 27863143

[33] Renner A , Burotto M , Rojas C . Immune checkpoint inhibitor dosing: can we go lower without compromising clinical efficacy? J Glob Oncol. 2019;5:1‐5. 10.1200/JGO.19.00142 PMC6690659 31348737

[34] Parchment RE , Doroshow JH . Pharmacodynamic endpoints as clinical trial objectives to answer important questions in oncology drug development. Semin Oncol. 2016;43:514‐525. 27663483 10.1053/j.seminoncol.2016.07.002 PMC5117459

[35] Ogungbenro K , Patel A , Duncombe R , Nuttall R , Clark J , Lorigan P . Dose rationalization of pembrolizumab and nivolumab using pharmacokinetic modeling and simulation and cost analysis. Clin Pharmacol Ther. 2018;103:582‐590. 28913853 10.1002/cpt.875

[36] Ogungbenro K , Patel A , Duncombe R , Clark J , Lorigan P . A rational approach to dose optimisation of pembrolizumab and nivolumab using cost analysis and pharmacokinetic modelling and simulation. Ann Oncol. 2016;27:vi370.

[37] Lala M , Li M , Sinha V , de Alwis D , Chartash E , Jain L . A six‐weekly (Q6W) dosing schedule for pembrolizumab based on an exposure‐response (E‐R) evaluation using modeling and simulation. J Clin Oncol. 2018;36:3062.30188790

[38] Dang TO , Ogunniyi A , Barbee MS , Drilon A . Pembrolizumab for the treatment of PD‐L1 positive advanced or metastatic non‐small cell lung cancer. Expert Rev Anticancer Ther. 2016;16(1):13‐20. 10.1586/14737140.2016.1123626 PMC4993158 26588948

[39] Li H , Yu J , Liu C , et al. Time dependent pharmacokinetics of pembrolizumab in patients with solid tumor and its correlation with best overall response. J Pharmacokinet Pharmacodyn. 2017;44:403‐414. 28573468 10.1007/s10928-017-9528-y

[40] Bach PB , Saltz LB . Raising the dose and raising the cost: the case of pembrolizumab in lung cancer. J Natl Cancer Inst. 2017;109(11). 10.1093/jnci/djx125 29059436

[41] Fernandes N . Economic effects of coronavirus outbreak (COVID‐19) on the world economy. SSRN Electron J. 2020. IESE Business School Working Paper No. WP‐1240‐E.

[42] Dingemans AMC , Soo RA , Jazieh AR , et al. Treatment guidance for patients with lung cancer during the coronavirus 2019 pandemic. J Thorac Oncol. 2020;15:1119‐1136. 32422364 10.1016/j.jtho.2020.05.001 PMC7227539

[43] Salako O , Okunade K , Allsop M , et al. Upheaval in cancer care during the COVID‐19 outbreak. Ecancermedicalscience. 2020;14:ed97. 10.3332/ecancer.2020.ed97 PMC7134578 32269597

